# Early-Stage Non-Small Cell Lung Cancer Stereotactic Body Radiation Therapy Outcomes in a Single Institution

**DOI:** 10.7759/cureus.21878

**Published:** 2022-02-03

**Authors:** Nathan P Doupnik, Khalid Hirmiz, Abdulkadir A Hussein, John Agapito, Ming Pan

**Affiliations:** 1 Department of Radiation Oncology, Windsor Regional Hospital, Windsor, CAN; 2 Department of Mathematics and Statistics, University of Windsor, Windsor, CAN; 3 Department of Medical Physics, Windsor Regional Hospital, Windsor, CAN

**Keywords:** non small cell lung cancer, stereostatic radiation therapy, nsclc stage i, stereotactic ablative radiation, retrospective comparative study

## Abstract

Introduction

The gold standard treatment of stage I non-small cell lung cancer (NSCLC) is surgical resection. For medically inoperable patients, stereotactic body radiation therapy (SBRT) can provide comparable local control (LC) and overall survival (OS). The objectives of this study are to determine the three-year LC and OS for SBRT compared to early-stage NSCLC patients treated with alternative radiation modalities at our institution.

Materials and methods

This retrospective study included a total of 139 consecutive patients who were diagnosed with stage I (T1-2 N0 M0) NSCLC and treated with radiation therapy at our institution between 2015 and 2020. Patient demographics and clinical data were obtained from chart reviews. Treatment subgroups were: SBRT (48Gy/4 or 60Gy/8), hypofractionation (60Gy/15), conventional fractionation (60Gy/30 or 50Gy/20), and palliative radiation (20Gy/5, 30Gy/10, or 40Gy/15). Kaplan-Meier curves were plotted for LC and OS. We also performed Cox’s proportional hazard regression analysis.

Results

The median patient age was 74 (range 52-91). The numbers of patients in each treatment subgroup were: SBRT (44), hypofractionation (78), conventional fractionation (8), and palliative (9). Differences in age, gender, and histopathological cell type between subgroups were not statistically significant. Metastatic progression was the most common outcome amongst treatment failures, followed by local recurrence and regional spread. Median post-treatment follow-up in months for each subgroup was: SBRT (20.2), hypofractionated (20.7), conventional fractionation (13.9), and palliative (14.4). Post-treatment three-year LC was found to be significantly better with SBRT (94%) versus hypofractionation (71%), conventional fractionation (80%), and palliative (71%). OS at three years were SBRT (67%), hypofractionation (59%), conventional fractionation (66%), and palliative (44%). As a whole, 72% (100/139) of patients had biopsy-proven NSCLC. Analysis showed biopsy status had no statistical significance with regards to LC or OS. Every 20 years of age had a 3.2x risk of death (95% CI: 1.425-7.268). Concerning the treatment modalities, there were significant differences for the hazard of death compared to SBRT: hypofractionation had 2.58x increased risk while palliative had 5.83x increased risk.

The proportion of patients who experienced post-treatment radiation pneumonitis or dermatitis were: SBRT (7%, 2%), hypofractionation (8%, 3%), conventional fractionation (13%, 25%), and palliative (0%, 0%), respectively. No patients who experienced grade III or higher toxicities were observed as defined by Common Terminology Criteria for Adverse Events (CTCAE).

Conclusion

Our experience confirms SBRT can provide durable three-year local control with a comparable rate of post-treatment complications versus other radiation modalities for early-stage NSCLC. SBRT appears to be non-inferior to hypofractionation with regards to three-year LC.

## Introduction

In 2020, lung cancer was the most commonly diagnosed cancer and the leading cause of cancer deaths in Canada. An estimated 30,000 Canadians were diagnosed with lung cancer, while around 21,000 died from it. More deaths occurred from lung cancer than breast, colorectal, and pancreatic cancers combined [1]. Poor prognosis is related to the fact that nearly half of all lung cancer patients present in stage IV. The most important modifiable risk factor continues to be smoking history; however, additional factors such as exposure to secondhand smoking, radon gas, and air pollution have been shown to play a role [2,3].

Lung cancers are classified based on biopsy interpretation, with the dominant type being non-small cell lung cancer (NSCLC), making up around 88%. NSCLC is further divided histologically as adenocarcinoma, squamous cell carcinoma, and large cell carcinoma. Of all patients with NSCLC, approximately 21% are present in stage I [1]. For these patients, surgery has traditionally been considered to have superior outcomes to radiation therapy for operable patients. In the last decade, however, there has been growing evidence to support the use of stereotactic body radiation therapy (SBRT) for both operable and non-operable patients [4]. Despite the lack of large-scale randomized controlled trials (RCTs), SBRT has seen a steady increase in its evidence base for use in early-stage NSCLC. International guidelines support the efficacy of SBRT in tumors < 5 cm, especially in patients with pre-existing pulmonary comorbidities [5]. For stage-I NSCLC, SBRT is known to achieve a durable three-year local control (LC) of > 90%, with three-year overall survival (OS) being less predictable due to variation in patient comorbidities [6].

Over the gradual adoption of SBRT, several concerns have been raised about its potential for increased side effects and toxicities, especially for centrally located tumors [7]. Adverse events such as the development of radiation-induced pneumonitis, dermatitis, and rib fractures are well documented [8,9]. Despite these concerns, the primary risk factor for the development of rib fractures in patients treated with SBRT is the proximity between the target site and adjacent rib(s) [10]. Fortunately, high biologically effective dose (BED) values such as BED >100 Gy have been associated with better outcomes for stage I/II NSCLC [11].

The results of pending randomized clinical trials such as the phase III Canadian OCOG-LUSTRE trial should provide further evidence for the efficacy and possible additional toxicity of SBRT versus other radiation modalities for early-stage NSCLC patients [12].

## Materials and methods

Study population and description

This retrospective study was conducted on 139 consecutive patients diagnosed with early-stage (stage I) NSCLC with no evidence of regional or distant metastasis who were treated with external beam radiation therapy at our institution (Windsor Regional Cancer Center) between January 2015 and June 2020 after obtaining Institutional Review Board (IRB) approval. Data collected on each patient included demographics, staging, pathological details, treatment characteristics, progression, and outcome. Data collection was completed on February 12, 2021.

All patients were presented at our multidisciplinary tumor board, including Surgery, Pulmonology, Medical Oncology, Radiology, Pathology, and Radiation Oncology. Any decision to proceed with radiation therapy without biopsy confirmation of disease was communicated and agreed upon in this forum, typically based on the predicted probability of malignancy (i.e., enlarging PET-avid lesion on serial scans) weighed against risks of biopsy.

Initial TNM staging was based on results of patient imaging studies, including computed tomography (CT) of the chest, abdomen, pelvis, and positron emission tomography (PET) scans. The overall staging was determined according to the American Joint Committee on Cancer, Eighth Edition guidelines [13]. Patients were eligible if target tumors were ≤ 5cm on the most recent imaging survey. Patients with more than one primary lung tumor without evidence of metastasis were also accepted. All patients were either deemed medically inoperable or declined surgery. No status was assigned to patients with hilar and mediastinal nodes ≤ 1cm with no abnormal uptake on FDG PET-CT scan.

Regimens used included: SBRT (48Gy/4 or 60Gy/8), hypofractionation (60Gy/15), conventional fractionation (60Gy/30 or 50Gy/20), and palliative radiation (20Gy/5, 30Gy/10, or 40Gy/15). For patients who received SBRT, a regimen of 60Gy in eight fractions was used for tumors whose gross tumor volume (GTV) was <1cm from great vessels or <2cm from the proximal bronchial tree, while 48Gy in four fractions was used for peripherally located primary tumors.

PET-CT was obtained in all patients for staging and target delineation. A 4D-CT simulation was performed. A GTV was generated, concerning all available phases of the 4D-CT scan, including the derived maximum intensity pixel (MIP) and average intensity pixel (CTAve) datasets. The GTV was expanded to a clinical target volume (CTV) or given the included motion information from the 4D-CT; this became the internal target volume (ITV). For SBRT cases, the GTV to CTV expansion was zero. This was further expanded to a planning target volume (PTV) according to technique, e.g., 5 mm for SBRT. All treatment planning and image-guided radiation therapy (IGRT) utilized the CTAve. Please refer to the NSCLC SBRT guidelines in the literature for further details [12,14].

Statistical analysis

Recurrence was documented as either local, regional, or metastatic progression. Local control (LC) was defined as no recurrence within the high-dose region of the primary target tumor. LC was determined for each tumor for patients who presented with multiple primary lung tumors. However, overall survival (OS) was calculated for each patient regardless of solitary or multiple tumor statuses. Radiation-induced toxicity was categorized according to the Common Terminology Criteria for Adverse Events (CTCAE) 5.0 [15].

Data were summarized using count (frequency) for categorical and median (range) for continuous variables. Time to event analyses was performed from the date of completion of radiotherapy and not from the date of tissue diagnosis or date of randomization as in other studies. Duration of follow-up was estimated using the reverse Kaplan-Meier method. OS and PFS were estimated using the Kaplan-Meier method and compared using the log-rank test. Biopsy versus non-biopsy differences was evaluated by unpaired two-tail Student’s t-test and two-tail Fisher’s exact test. Multivariate analysis using Cox’s proportional hazard model was performed to determine whether baseline patient demographics impacted survival. P values of ≤ 0.05 were of statistical significance. In an analysis involving parametric and non-parametric statistical tests, significance was assigned if p ≤ 0.05 for both.

## Results

Baseline patient data

Amongst all 139 patients included, median follow-up was 19.7 months (range 0-66.8 months). The median age at the start of treatment was 74 (range 52-91) years old. The median pack-years of smoking was 45. Breakdown of target lesion size by AJCC 8th edition T-stage was as follows: T1 (89.9%) and T2 (10.1%). The median lesion size was 2.1cm. The numbers of patients in each treatment subgroup were: SBRT (44), hypofractionation (78), conventional fractionation (8), and palliative (9). Median post-treatment follow-up in months for each subgroup was: SBRT (20.2), hypofractionated (20.7), conventional fractionation (13.9), and palliative (14.4). Baseline patient characteristics are summarized in Table 1.

**Table 1:** Baseline patient characteristics n: number, %: percentage, pk/yr: number of smoking pack-year equivalents, PFTs: pulmonary function tests, FEV 1: forced expiratory volume in one second, DLCO: diffusing capacity of carbon monoxide gas, ECOG: Eastern Cooperative Oncology Group, AC: adenocarcinoma, SCC: squamous cell carcinoma, Gy: gray, fx: number of fractions,

Gender	n	%
Male	65	46.8
Female	74	53.2
Age (years)	Median	Range
	74	(52-91)
Smoking		
<10 pk/yr	15	10.8
10-39 pk/yr	33	24.8
40 or more pk/yr	82	61.7
Unknown pk/yr	9	6.8
PFTs (% predicated value)	Median	Range
FEV 1	71%	(18-130)
DLCO	50%	(14-131)
ECOG Score	n	%
0	38	27.3
1	42	30.2
2	14	10.1
3	3	2.2
Unknown	42	30.2
Stage	n	%
T1	125	89.9
T2	14	10.1
T3	0	0
Size (cm)	Median	Range
	2.1	(0.8-4.9)
Histology	n	%
AC	60	43.2
SCC	31	20.1
Other Type	20	22.3
Indeterminate / No Biopsy	28	14.4
Dose / # fractions	n	%
60 Gy/ 8 fx	6	4.3
48 Gy / 4 fx	38	27.3
60 Gy / 15 fx	78	56.1
60 Gy / 30 fx	8	5.8
50 Gy / 20 fx	1	0.7
40 Gy / 15 fx	5	4.3
30 Gy / 10 fx	1	0.7
20 Gy / 5 fx	2	1.4

Multivariate analysis revealed no significant difference between the proportions of gender, age, and baseline pulmonary function test performance (FEV1 and DLCO) between the four subgroups, as shown in Table 2. 

**Table 2:** Analysis of baseline patient demographic data n: number, Col%: column percentage, AC: adenocarcinoma, SCC: squamous cell carcinoma, ECOG: Eastern Cooperative Oncology Group, FEV1: forced expiratory volume in one second, DLCO: diffusing capacity of carbon monoxide * Calculated by ANOVA for numerical covariates and chi-square test for categorical covariates. ** Calculated by the Kruskal-Wallis test for numerical covariates and Fisher's exact test for categorical covariates. *** Patients with unknown smoking history were excluded from this calculation.

Covariate	Statistics	Level	SBRT N=44	Palliative N=9	Hypofractionated N=78	Conventional Fractionation N=8	Parametric P-value*	Non-Parametric P-value**
Gender	N (Col %)	F	24 (54.55)	2 (22.22)	44 (56.41)	4 (50)	0.277	0.284
	N (Col %)	M	20 (45.45)	7 (77.78)	34 (43.59)	4 (50)		
Age	N		44	9	78	8	0.202	0.310
	Mean		73.59	76.22	74.68	68.5		
	Median		72.5	77	74.5	65.5		
Stage	N (Col %)	T1	41 (93.18)	2 (22.22)	56 (71.79)	4 (50)	< .001>	< .001>
	N (Col %)	T2	3 (6.82)	7 (77.78)	22 (28.21)	4 (50)		
Biopsy	N (Col %)	No	21 (47.73)	2 (22.22)	15 (19.23)	1 (12.5)	0.006	0.006
	N (Col %)	Yes	23 (52.27)	7 (77.78)	63 (80.77)	7 (87.5)		
Cell Type	N (Col %)	AC	17 (38.64)	3 (33.33)	39 (50)	1 (12.5)	0.141	0.114
	N (Col %)	Other	10 (22.73)	1 (11.11)	8 (10.26)	1 (12.5)		
	N (Col %)	SCC	6 (13.64)	4 (44.44)	17 (21.79)	4 (50)		
	N (Col %)	Unknown	11 (25)	1 (11.11)	14 (17.95)	2 (25)		
ECOG	N (Col %)	0	18 (43.9)	0 (0)	17 (37.78)	3 (42.86)	< .001>	0.005
	N (Col %)	1	19 (46.34)	0 (0)	19 (42.22)	4 (57.14)		
	N (Col %)	2	3 (7.32)	2 (50)	9 (20)	0 (0)		
	N (Col %)	3	1 (2.44)	2 (50)	0 (0)	0 (0)		
Smoking (pack years) ***	N		42	7	73	8	0.021	0.064
	N (Unknown)		2	2	5	0		
	Mean		47.69	49.29	48.69	21.44		
	Median		50	50	45	19		
FEV1	N		42	8	73	8	0.138	0.108
	Median		0.63	0.61	0.8	0.74		
DLCO	N		35	4	64	8	0.590	0.884
	Median		0.49	0.45	0.51	0.52		

Disease outcomes and treatment failures

Post-treatment three-year LC was best for SBRT (94%), versus hypofractionation (71%), conventional fractionation (80%), and palliative (71%). OS at three years were SBRT (67%), hypofractionation (59%), conventional fractionation (66%), and palliative (44%). Of all patients, the patterns of relapse included 21 local failures (15.1%), six regional failures (4.3%), and 32 who had metastatic progression (23.0%). Patients treated with SBRT had a similar distribution of failures versus patients treated with other modalities. This data is summarized in Table 3.

**Table 3:** Primary outcomes and failures by treatment subgroup n: number, SBRT: stereotactic body radiation, LC: local control, OS: overall survival, Col%: column percentage

Treatment	SBRT (N=44)	Hypofractionation (N=78)	Conventional Fractionation (N=8)	Palliative (N=9)
Three Year LC	94%	71%	80%	71%
Three Year OS	67%	59%	66%	44%
Local Recurrence, N (Col%)	2 (4.5%)	14 (17.9%)	2 (25.0%)	3 (33.3%)
Regional Recurrence, N (Col%)	1 (2.3%)	4 (5.1%)	1 (12.5%)	0 (0.0%)
Metastatic Progression, N (Col%)	5 (11.4%)	23 (29.5%)	2 (25.0%)	4 (44.4%)

**Figure 1:**
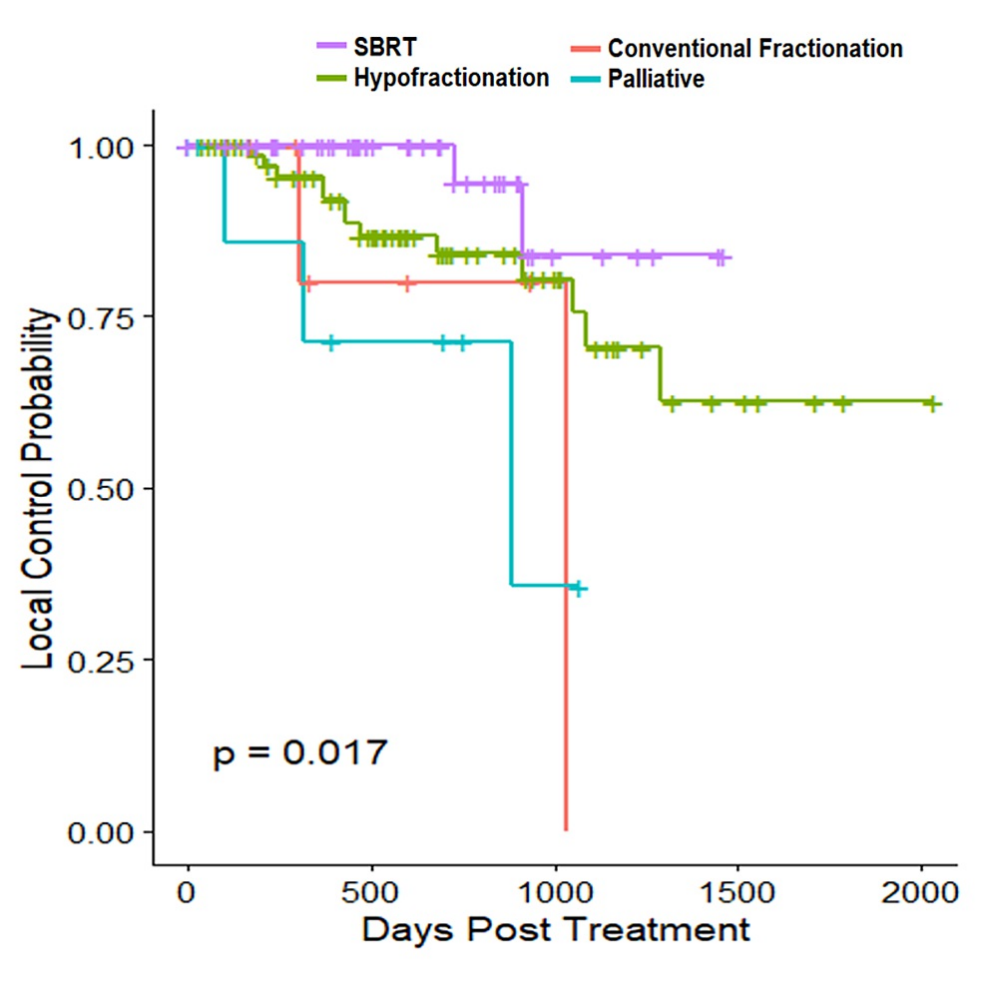
Primary tumor local control by subgroup SBRT: stereotactic body radiation therapy

Figure 1 below shows the Kaplan-Meier curve for LC. 

**Figure 2:**
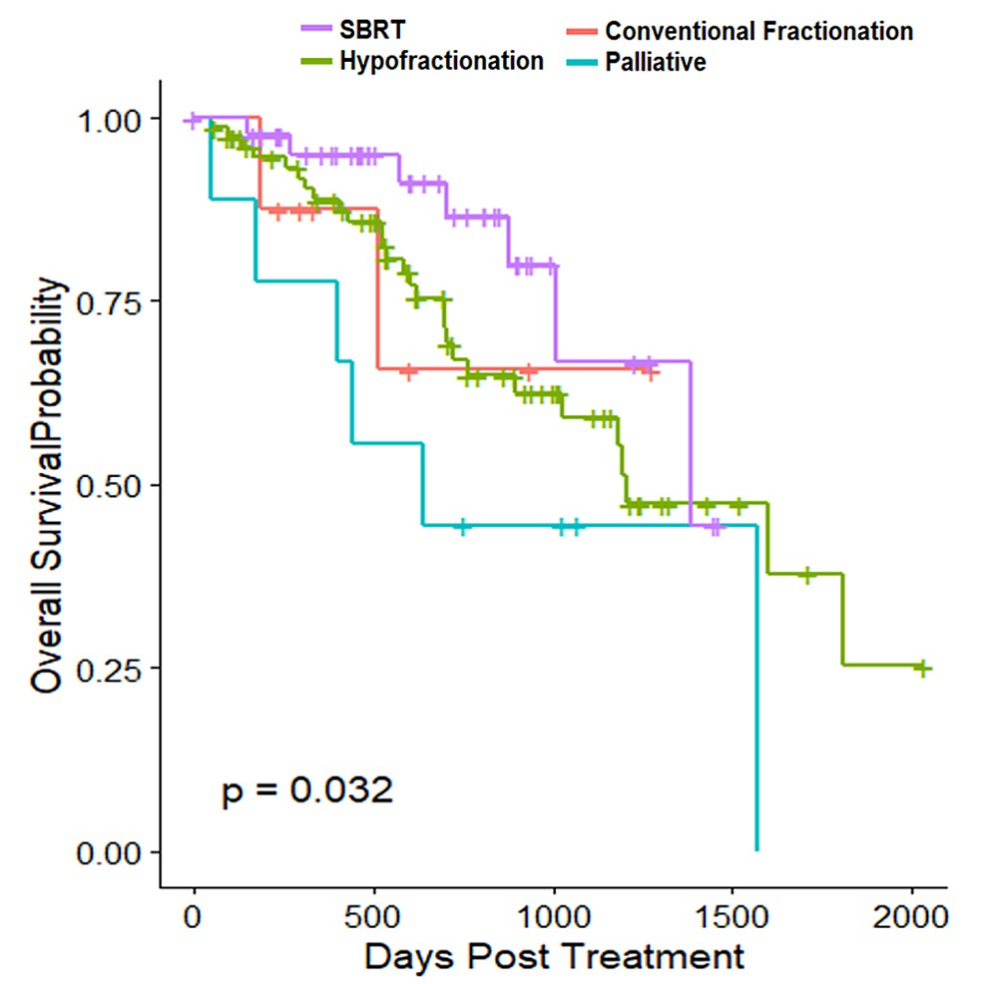
Overall survival by subgroup SBRT: stereotactic body radiation therapy

Figure 2 below shows the Kaplan-Meier curve for OS. 

With regards to outcomes observed, three-year LC was highest for SBRT patients (94%) and was statistically significant compared to palliative (p=0.021, Sidak’s pairwise comparison). Compared to SBRT, hazard ratio (HR) for risk of local recurrence were as follows: hypofractionation: 3.127 (95% CI 0.70-13.98) and conventional fractionation: 6.51 (95% CI 0.86- 49.21). The only statistically significant difference in three-year OS was between SBRT and palliative (HR 4.286, 95% CI 1.42-12.93). Additionally, every 20 years of age had a 3.2x risk of death (95% CI: 1.425-7.268).

Treatment-related toxicities

Of all patients included in the study, 5.8% developed Grade 1 toxicity consisting of asymptomatic pneumonitis or mild dermatitis. 5.0% of patients developed Grade 2 toxicity corresponding to pneumonitis requiring medical treatment and moderate moist dermatitis. No rib fractures or Grade 3 or higher toxicities were observed in accordance with the CTCAE v5.0 [15]. This data is summarized in Table 4.

**Table 4:** Observed toxicity profile SBRT: stereotactic body radiation therapy, n: number, %: percent of group

	Grade 1		Grade 2		Grade 3-5		Total	
	SBRT	All Patients	SBRT	All Patients	SBRT	All Patients	SBRT	All Patients
Pneumonitis, n (%)	3 (6.8%)	5 (3.6%)	0	5 (3.6%)	0	0	3 (6.8%)	10 (7.2%)
Dermatitis, n (%)	1 (2.3%)	3 (2.2%)	0	2 (1.4%)	0	0	1 (2.3%)	5 (3.6%)
Rib Fractures	0	0	0	0	0	0	0	0

Biopsy versus non-biopsy

The biopsy status of the subgroups was as follows, SBRT (23 out of 44), Hypofractionation (63 out of 78), conventional fractionation (7 out of 8), and palliative (7 out of 9). Statistical analysis revealed significant differences (p<0.05) in age, FEV1%, and mean tumor size. These results are summarized in Table 5.

**Table 5:** Significance of biopsy versus non-biopsy n: number, SD: standard deviation, FEV1: forced expiratory volume in one second, DLCO: diffusing capacity of carbon monoxide

	Male (n)	Female (n)	Mean age years (+/- SD)	Smoking Mean Pack-years (+/- SD)	Mean FEV1 % predicted (+/- SD)	Mean DLCO % predicted (+/- SD)	T1 Stage, n	T2 Stage, n	Mean Tumor Size, cm (+/- SD)
No Biopsy or indeterminate	19	20	71 (7.64)	54.21 (27.32)	61.95 (22.44)	49.81 (17.53)	38	1	1.96 (0.82)
Biopsy	46	54	75.28 (8.46)	43.51 (30.17)	77.28 (22.07)	54.49 (19.47)	87	13	2.59 (1.03)
p-Value	0.8506		0.0068	0.0592	0.00005	0.2407	0.1126		0.0009

**Figure 3:**
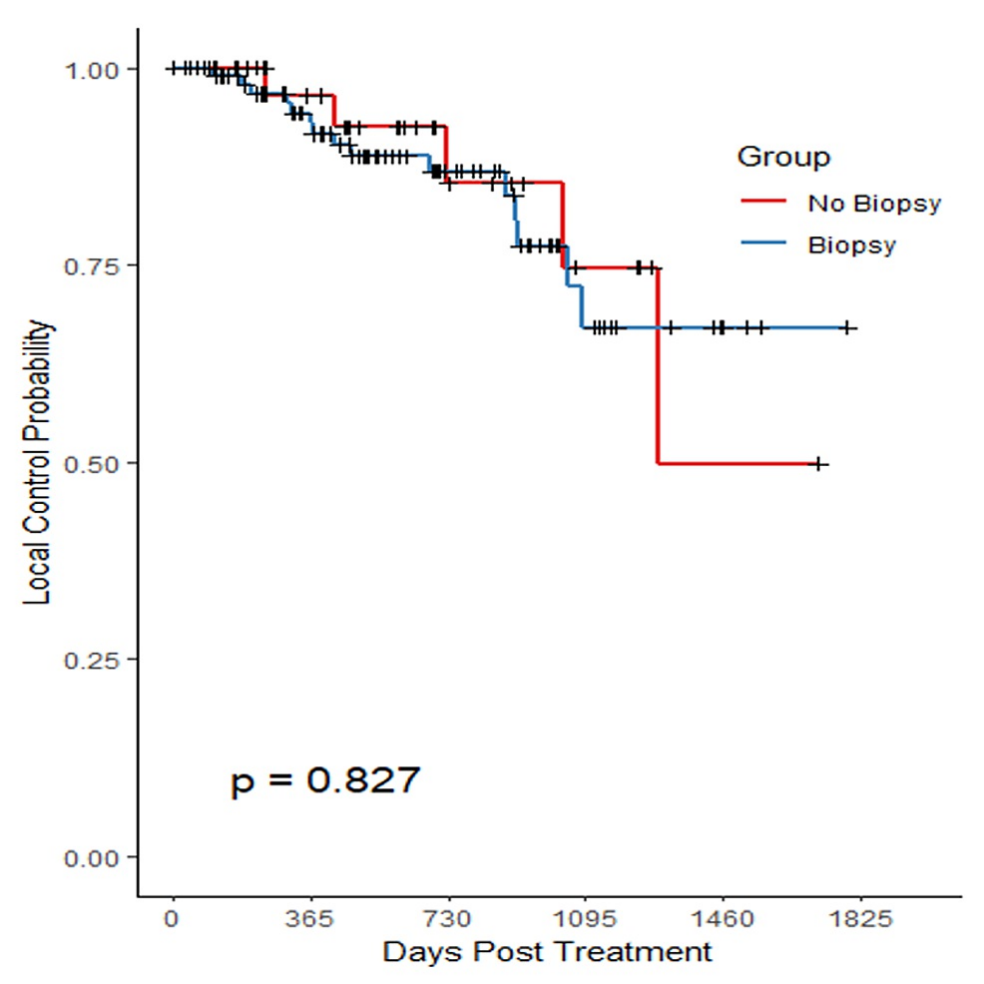
Biopsy effect on local control

With regards to LC, there was no statistical difference between groups. This is reflected by the Kaplan-Meier curve in Figure 3.

**Figure 4:**
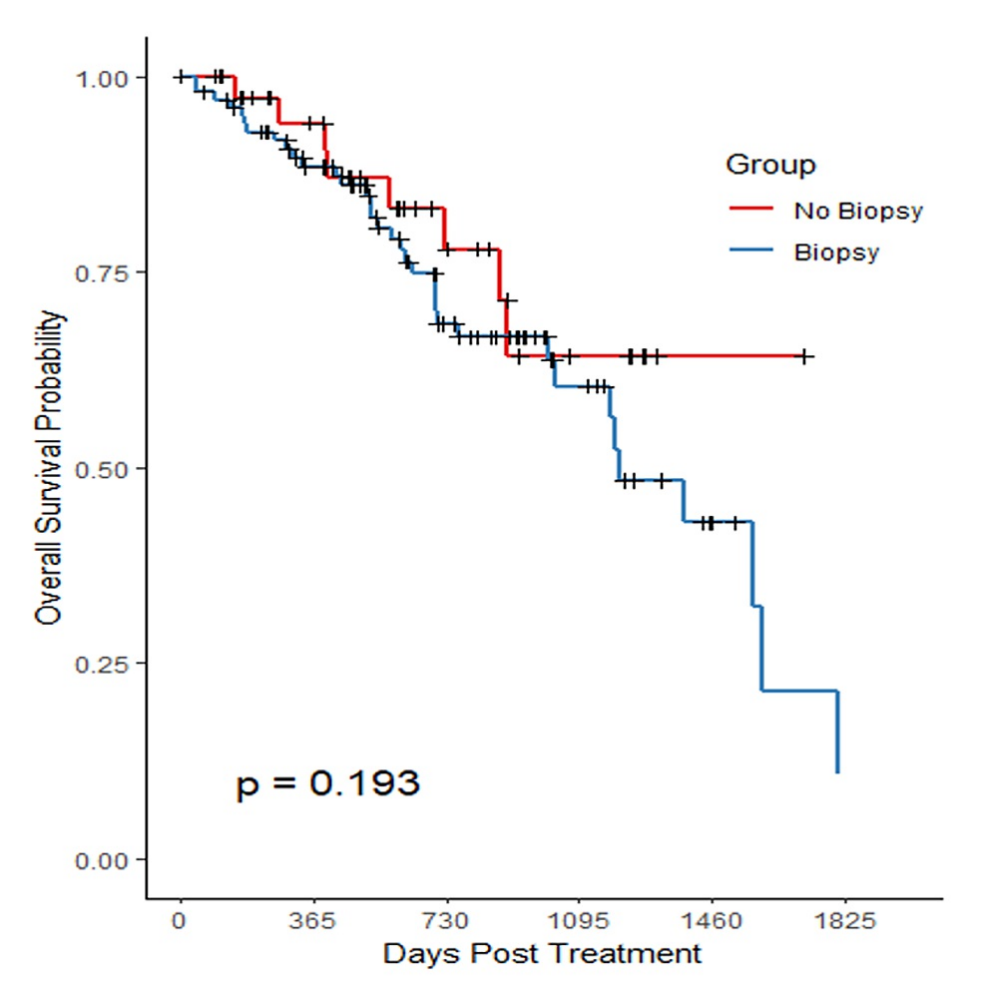
Biopsy effect on overall survival

With regards to OS, there was no statistical difference between groups. This is reflected by the Kaplan-Meier curve in Figure 4. 

## Discussion

In the last decade, SBRT has become more common in its use in early-stage NSCLC. Although there is some evidence in the literature to support such use of SBRT for both operable and non-operable NSCLC patients, there are no existing large-scale RCTs to show significant benefit over conventional radiotherapy. We conducted this retrospective study to evaluate the outcome of SBRT versus other radiation modalities in our institution.

**Figure 5:**
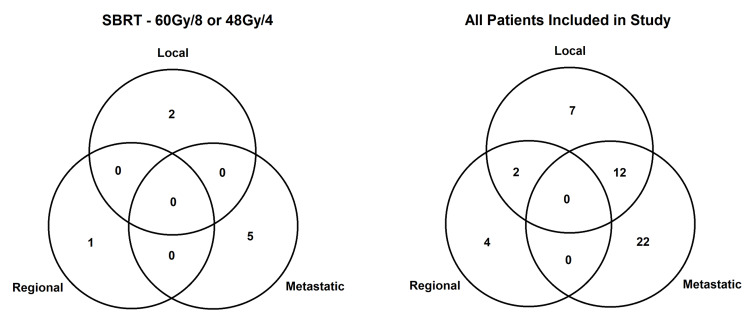
Patterns of treatment failure SBRT: stereotactic body radiation therapy, Gy: gray, /: radiation dose given in number of fractions

For patients treated with SBRT, the three-year LC of 94% is consistent with values reported in the literature for early-stage NSCLC [16-20]. In comparison, patients treated with hypofractionation had a three-year LC of 71%. This difference approached statistical significance (95%CI 0.86-4.71). Comparing three-year OS, SBRT did not offer a statistically significant benefit versus hypofractionation. We favour LC over OS to be our primary endpoint because many patients died of non-cancer causes (pneumonia, [chronic inflammatory lung disease] COPD exacerbations, cardiovascular complications, etc.), which makes any small survival benefit difficult to appreciate. Similar patterns of treatment failure were observed regardless of the radiation treatment subgroup, with distant metastatic progression being the dominant failure outcome, as shown in Figure 5.

Previous case reports of SBRT in the earlier years showed some severe toxicities [21-23]; however, none of our 139 consecutive patients had post-radiation treatment toxicities of grade III or higher. SBRT had similar rates of radiation dermatitis and pneumonitis versus other subgroups. This could be due to the patient selection process where we only offer conventional radiotherapy if their SBRT plan failed to pass normal organ-at-risk dose criteria on Dose-Volume-Histogram (DVH). Our results support the hypothesis that SBRT is non-inferior to hypofractionation for early-stage inoperable NSCLC patients.

One of the limitations of our study is that only 72% (N=100) were biopsy-proven. Percutaneous transthoracic lung biopsy has not been shown to increase the risk of pleural cancer recurrence [24]. However, all patients with enlarging PET-avid lesions on serial scans were presented at our multidisciplinary tumor board, and the decision to proceed with radiation therapy without tissue diagnosis was weighed against the risks of biopsy. This is consistent with the patient enrollment criteria in other large RCTs such as OCOG-LUSTRE [12]. Other reasons for non-biopsy in our patients included patient refusal and previous failed biopsy attempts. In the literature, the major risk of trans-thoracic or trans-bronchial biopsy is pneumothorax, with an average risk of 20% (range 9-54%) [25]. The risk is higher in NSCLC patients who are unfit for surgery due to underlying pulmonary comorbidities such as COPD/emphysema. There is a growing trend to treat early-stage suspected NSCLC patients who are unfit for surgery with SBRT without prior diagnostic lung biopsy [26].

There was a statistically significant difference between biopsy status in our treatment subgroups. Analysis from the data in Figures 3, 4; however, suggests that biopsy status had no statistically significant impact on LC or OS. Histological analysis revealed that adenocarcinoma cases outnumbered squamous cell carcinoma (SCC), approximately 2:1, which is consistent with the distribution reported in the literature [1]. Biopsied patients had highly statistically significant differences (p<0.001) about larger tumor size and higher FEV1 values compared to non-biopsied patients, reflecting clinical judgement to biopsy larger lesions in fitter patients.

Other limitations included the non-randomization of patients and small sample sizes in the conventional and palliative radiation subgroups. There was also statistical significance in the proportion of T1 versus T2 tumors between the subgroups with SBRT and hypofractionation, with the latter having a larger burden of T2 tumors, which is associated with worse LC and OS [27]. Another possible selection bias in favour of SBRT is the fact that fitter patients were more likely to make it through the demanding immobilization process and 4D-CT simulation planning. In our study, the median follow-up time for patients treated with SBRT was 20.2 months, which may have been too short to observe complications such as rib fractures. Related studies have reported a median of 22 months for rib fractures from SBRT treatment [28].

To date, evidence from the SPACE trial has suggested that while SBRT is more convenient to patients, it offers no significant LC or OS benefit [29]. The phase-III TROG-CHISEL trial is ongoing, which hopes to further expand on the evidence for the use of SBRT versus conventional fractionation [30]. We anticipate the results from the Canadian OCOG-LUSTRE trial will clarify the benefit of SBRT versus hypofractionation for early-stage inoperable NSCLC patients [12].

## Conclusions

Our experience confirms SBRT can provide durable local control with a comparable rate of post-treatment complications versus other radiation modalities for early-stage NSCLC. Further evidence from large randomized controlled trials is needed to establish the possible superiority of SBRT versus hypofractionated and conventional fractionated modalities.
